# Mindfulness and athlete burnout among olympic combat sport athletes: the mediating role of athlete self-compassion and the moderating role of emotion regulation

**DOI:** 10.3389/fpsyg.2026.1941380

**Published:** 2026-09-03

**Authors:** Zülbiye Kaçay, Emrah Seçer, Özkan Işık, Yunus Şahinler, Laurentiu-Gabriel Talaghir, Mariana Constantinescu, Cristina-Corina Benţea, Teodora Mihaela Iconomescu

**Affiliations:** 1 Faculty of Sport Sciences, Çanakkale Onsekiz Mart University, Çanakkale, Türkiye; 2 Faculty of Sport Sciences, Erzincan Binali Yildirim University, Erzincan, Türkiye; 3 Faculty of Sport Sciences, Balikesir University, Balikesir, Türkiye; 4 Faculty of Sport Sciences, Istanbul Gelişim University, Istanbul, Türkiye; 5 Faculty of Physical Education and Sport, Dunarea de Jos University of Galati, Galati, Romania; 6 Faculty of Education Sciences, Dunarea de Jos University of Galati, Galati, Romania

**Keywords:** athlete burnout, athlete self-compassion, combat sports, emotion regulation, sport mindfulness

## Abstract

**Introduction:**

The present study aimed to examine the effect of sport mindfulness on athlete burnout among Olympic combat sport athletes and to investigate the mediating role of athlete self-compassion and the moderating role of emotion regulation in this relationship.

**Methods:**

This study employed a cross-sectional correlational research design and included 379 athletes (279 men and 100 women) actively competing in Olympic combat sports. Data were collected using the Mindfulness Inventory for Sport, the Athlete Burnout Questionnaire, the Self-Compassion Scale–Athlete Version-Short Form and the Athlete Emotion Regulation Scale. The construct validity of the measurement instruments was evaluated using confirmatory factor analysis. Relationships among the study variables were examined using Pearson correlation analysis. The proposed hypotheses were tested using the PROCESS macro, specifically Models 4, 1, and 14.

**Results:**

The findings indicated that sport mindfulness positively and significantly predicted athlete self-compassion (B = 0.155, *p* < 0.001). Athlete self-compassion, in turn, was found to have a significant negative effect on athlete burnout (*B* = −0.798, *p* < 0.001). Furthermore, the interaction between athlete self-compassion and emotion regulation significantly predicted athlete burnout (*B* = −0.060, *p* < 0.001). The moderated mediation analysis demonstrated that emotion regulation significantly moderated the indirect association between sport mindfulness and athlete burnout (Index = −0.009). Examination of the conditional indirect effects revealed that the burnout-reducing effect of sport mindfulness through athlete self-compassion was stronger among athletes with higher levels of emotion regulation.

**Conclusion:**

The findings suggest that the association between sport mindfulness and athlete burnout cannot be explained solely by a direct relationship. Rather, this relationship operates through athlete self-compassion and varies according to athletes' levels of emotion regulation. These findings underscore the importance of integrating mindfulness-based skills, self-compassion development, and emotion regulation capacity into interventions designed to enhance psychological resilience and prevent burnout among combat sport athletes.

## Introduction

Olympic combat sports—including wrestling, boxing, judo, taekwondo, karate, and fencing—require athletes not only to demonstrate exceptional physical capacity and technical proficiency but also to effectively manage their psychological resources within highly competitive environments (Andreato et al., 2022). The one-on-one nature of these disciplines, coupled with minimal tolerance for errors, the strong dependence of competition outcomes on individual performance, and persistent performance pressure, exposes athletes to chronic psychological stress (McLoughlin et al., 2022). Moreover, demanding training loads, congested competition schedules, weight management associated with weight categories, injury risk, and expectations for success at both national and international levels place substantial demands on athletes' cognitive and emotional resources in addition to their physical capacities (Burke et al., 2021). When these demands persist over time and exceed the available psychological resources, the risk of athlete burnout may increase.

Athlete burnout is widely recognized as a multidimensional psychological syndrome characterized by emotional and physical exhaustion, a reduced sense of accomplishment, and sport devaluation, all of which adversely affect sustainable performance, sport commitment, and career longevity (Olsson et al., 2025). Nevertheless, considerable variation in burnout levels among athletes exposed to comparable training loads and competitive conditions suggests that burnout cannot be explained solely by external demands (Madigan et al., 2021). Rather, psychological resources and self-regulatory processes are also likely to play a critical role in its development (Gayretli et al., 2026). Accordingly, identifying the psychological mechanisms that may protect against burnout in Olympic combat athletes, as well as the individual conditions under which these mechanisms operate more effectively, represents an important research priority for promoting sustainable athletic performance and enhancing athletes' psychological wellbeing.

### Sport mindfulness and athlete burnout

Mindfulness is defined as the capacity to attend to present-moment experiences with openness, awareness, and a non-judgmental attitude (Kabat-Zinn, 2003; Lindsay and Creswell, 2017). Within the field of sport psychology, mindfulness is regarded as an important psychological resource that enables athletes to sustain attentional focus during performance, regulate emerging thoughts and emotions, and respond more adaptively to stressors encountered in competitive settings (Birrer et al., 2012; Nien et al., 2023). In elite sport, where athletes are continually exposed to high performance expectations, competitive pressure, fear of failure, and ongoing evaluation, the effective management of psychological resources is essential for maintaining sustainable performance.

One of the primary mechanisms through which mindfulness reduces stress-related adverse outcomes is by encouraging individuals to approach their experiences with greater acceptance and balance rather than reacting automatically or impulsively. Accordingly, mindfulness has been suggested to be a trainable psychological capacity that can be enhanced through mindfulness-based interventions, which have recently become one of the most widely investigated psychological and behavioral approaches in both clinical and sport settings (Zhao et al., 2026). According to the Mindfulness-to-Meaning Theory, mindfulness facilitates more adaptive cognitive appraisals of stressful events by reducing threat perception and promoting more constructive meaning-making processes (Garland et al., 2015). This theoretical perspective provides an important framework for understanding how athletes develop psychological flexibility when confronted with failure, performance pressure, and competition-related stress.

Growing empirical evidence indicates that mindfulness is closely associated with lower burnout, reduced stress, and enhanced psychological wellbeing among athletes (Kelemen et al., 2025; Wang et al., 2025). For example, Zhang et al. (2021) reported that elite athletes with higher levels of mindfulness experienced lower athlete burnout and greater psychological wellbeing. Likewise, Secer et al. (2025) demonstrated that greater mindfulness contributed to increased life satisfaction through its positive effect on psychological resilience. Similarly, mindfulness-based interventions have been shown to alleviate stress symptoms while strengthening psychological resilience in athletic populations (Noetel et al., 2019). Recent meta-analytic evidence has further confirmed that mindfulness-based interventions improve performance-related psychological outcomes and facilitate more effective stress management among athletes (Laulan et al., 2026).

Burnout is characterized by emotional, cognitive, and physical exhaustion accompanied by symptoms such as fear of failure (Gustafsson et al., 2018). Athlete burnout, in particular, is recognized as a multidimensional psychological syndrome resulting from chronic sport-related stress and is characterized by emotional and physical exhaustion, a reduced sense of accomplishment, and sport devaluation (Raedeke and Smith, 2001; Taskin and Taskin, 2026). Contemporary perspectives suggest that athlete burnout cannot be attributed solely to excessive training loads or physical fatigue; rather, athletes' appraisal of stressors and the psychological resources available to them play a pivotal role in the development of burnout (Biçer Baikoglu et al., 2026). According to Conservation of Resources Theory, individuals experience stress when they perceive that their personal resources are threatened or depleted (Hobfoll, 1989). Within this framework, mindfulness can be viewed as a valuable personal resource that enables athletes to preserve their psychological resources when facing stressful experiences.

Olympic combat sports present a particularly high-risk context for burnout because athletes are continually exposed to intense competition, weight management demands, injury risk, and sustained performance pressure. Competitive sport has also been described as an environment characterized by continuous monitoring, comparison, and evaluation of training and performance (Zhang, 2026). Considering the well-established influence of psychological skills such as mindfulness on athletic performance, investigating its potential protective role against athlete burnout appears particularly important (Duyan et al., 2026a). Nevertheless, existing research has predominantly focused on the direct association between mindfulness and burnout, while relatively little attention has been devoted to identifying the psychological mechanisms through which this relationship operates. In this regard, athlete self-compassion emerges as a promising mechanism that may help explain how mindfulness contributes to lower levels of athlete burnout.

### The mediating role of athlete self-compassion

Self-compassion refers to an individual's capacity to respond to experiences of failure, inadequacy, or stress with understanding, kindness, and support rather than harsh self-criticism (Neff, 2003). It comprises three fundamental components—self-kindness, common humanity, and mindfulness—which together facilitate more balanced psychological responses to adverse experiences (Neff and Germer, 2022). Within the sporting context, self-compassion is considered an important psychological resource that helps athletes reduce destructive self-criticism following performance errors, competitive setbacks, and intense performance pressure, thereby promoting psychological wellbeing, adaptive functioning, and sustainable performance (Wang et al., 2025; Terzioglu and Çakir-Çelebi, 2025). Higher levels of self-compassion have also been associated with healthier self-evaluation, greater capacity to learn from mistakes, and a tendency to perceive failure as a universal human experience rather than as a personal deficiency (Mosewich et al., 2013). Accordingly, self-compassionate individuals are expected to engage in less self-criticism and exhibit fewer symptoms of burnout (Amemiya and Sakairi, 2020). Within competitive sport, constant performance evaluation and intense competition may lead athletes to interpret failures as personally threatening experiences, thereby increasing self-critical tendencies. When prolonged, these experiences may elevate the risk of athlete burnout. From the perspective of Conservation of Resources Theory, individuals strive to preserve their psychological resources, whereas the loss or threatened loss of these resources accelerates stress and burnout processes (Hobfoll, 1989). Consequently, self-compassion may function as a protective psychological resource that helps athletes preserve their psychological wellbeing when confronted with stressful sporting experiences.

The association between mindfulness and self-compassion can be understood through self-compassion theory and mindfulness-based perspectives. Mindfulness reduces excessive identification with negative thoughts and emotions, thereby fostering a more accepting and compassionate attitude toward oneself (Neff, 2003). Athletes with higher levels of mindfulness are therefore expected to evaluate performance setbacks more objectively and adopt more supportive self-attitudes following mistakes. Accumulating evidence indicates that self-compassion is an important psychological resource associated with lower stress, enhanced psychological wellbeing, and reduced burnout among athletes. Killham et al. (2018) demonstrated that athlete self-compassion is associated with lower performance anxiety and fewer adverse psychological outcomes. More recent studies have further shown that self-compassion enhances psychological resilience, mitigates the negative effects of stressful experiences, and serves as a protective factor against athlete burnout (Cormier et al., 2025). In addition, mindfulness-based processes have been shown to promote psychological adjustment by strengthening self-compassion (Neff and Germer, 2022).

Taken together, these findings suggest that the influence of mindfulness on athlete burnout is unlikely to operate solely through a direct pathway. Rather, the compassionate attitude athletes develop toward themselves may represent a key psychological mechanism underlying this relationship. Accordingly, athlete self-compassion was conceptualized as the mediating variable linking mindfulness and athlete burnout in the present study. Nevertheless, self-compassion alone may not fully account for this relationship, as athletes may differ in their ability to effectively utilize their psychological resources under stressful performance conditions. In this respect, emotion regulation represents an important individual difference variable that enables athletes to manage their emotional responses to stress, failure, and performance pressure and may therefore influence the effectiveness of this psychological process.

### The moderating role of emotion regulation

Emotion regulation is a fundamental psychological capacity that refers to the processes through which individuals monitor, evaluate, and modify their emotional responses (Gross, 1998). According to the Process Model of Emotion Regulation, emotions are regulated through a range of strategies, particularly cognitive reappraisal and expressive suppression (Gross, 2015). Effective emotion regulation requires individuals not only to recognize their own emotions but also to understand the emotional experiences of others and determine appropriate responses to different emotional situations. It is further characterized by the capacity to regain psychological energy, restore motivation following adverse experiences, and maintain a focus on future achievement despite setbacks (Kaya and Onag, 2024). Within sport settings, emotion regulation skills play a crucial role in enabling athletes to respond more adaptively to performance pressure, competitive stress, and experiences of failure (Tamminen et al., 2025). For elite athletes, demanding training schedules and consistently high performance expectations create circumstances that require continuous emotional regulation. When negative emotions are not managed effectively, accumulated stress may deplete psychological resources and increase vulnerability to athlete burnout (Gross, 2015). Conversely, effective emotion regulation enables athletes to appraise stressful experiences more constructively, maintain psychological adjustment (Duyan et al., 2026b), and promote overall mental wellbeing (Zekioğlu et al., 2024).

The moderating role of emotion regulation in the association between athlete self-compassion and burnout can be understood within the framework of Conservation of Resources Theory. According to this theory, psychological resources function as protective assets that help individuals cope with stress. However, the effectiveness of these resources depends largely on an individual's capacity to regulate and utilize them appropriately (Hobfoll, 1989). From this perspective, although self-compassion represents an important psychological resource for athletes, emotion regulation may determine the extent to which this resource can be effectively mobilized under demanding sport conditions.

Recent evidence suggests that emotion regulation is closely associated with psychological resilience, stress management, and burnout among athletes (Mei et al., 2025; Zuo and Bai, 2025). In particular, adaptive emotion regulation strategies, such as cognitive reappraisal, have been linked to more favorable psychological outcomes, whereas maladaptive regulation strategies appear to undermine psychological adjustment (Ford and Troy, 2019). Accordingly, individual differences in emotion regulation may strengthen or weaken the protective effect of athlete self-compassion against burnout. Therefore, in the present study, emotion regulation was conceptualized as a moderator of the relationship between athlete self-compassion and athlete burnout. Furthermore, the study examined whether the indirect effect of sport mindfulness on athlete burnout through athlete self-compassion varied according to athletes' levels of emotion regulation within a moderated mediation framework.

Accumulating evidence suggests that sport mindfulness is associated with the ways athletes appraise stressful experiences, adapt psychologically, and experience burnout. Nevertheless, further research is needed to clarify the psychological mechanisms underlying the relationship between sport mindfulness and athlete burnout, as well as the individual characteristics that may influence this association. This issue is particularly relevant in Olympic combat sports, where athletes are exposed to intense performance pressure, demanding training loads, and continuous self-regulation requirements (Gülsoy and Erhan, 2024). Under such conditions, examining psychological resources together with the factors that influence their effective utilization may contribute to a more comprehensive understanding of the burnout process. Accordingly, the present study investigated the mediating role of athlete self-compassion and the moderating role of emotion regulation in the relationship between sport mindfulness and athlete burnout among Olympic combat athletes. In addition, emotion regulation was proposed as an individual characteristic that may influence the strength of the association between athlete self-compassion and athlete burnout. Based on this conceptual framework, the following hypotheses were formulated:

**H1:** Sport mindfulness is negatively and significantly associated with athlete burnout.
**H2:** Athlete self-compassion mediates the relationship between sport mindfulness and athlete burnout.
**H3:** Emotion regulation moderates the relationship between athlete self-compassion and athlete burnout.
**H4:** The indirect effect of sport mindfulness on athlete burnout through athlete self-compassion varies according to the level of emotion regulation.

## Method

### Research design

**Figure 1:**
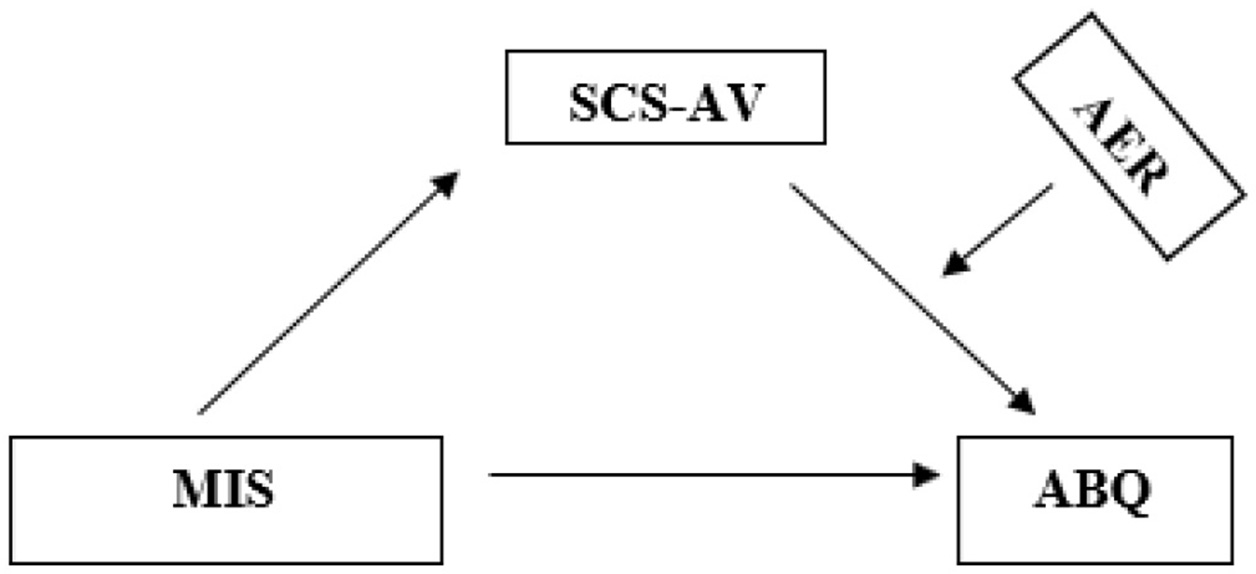
Research model. MIS, Mindfulness Inventory for Sport; SCS-AV, Self-Compassion Scale–Athlete Version–Short Form; ABQ, Athlete Burnout Questionnaire; AER, Athlete Emotion Regulation Scale.

This study was designed using a quantitative correlational research design to examine the effect of sport mindfulness on athlete burnout among combat sport athletes and to investigate the mediating role of athlete self-compassion and the moderating role of emotion regulation in this relationship. Correlational research is an appropriate design for identifying the direction and magnitude of relationships among two or more variables, examining the structural associations between variables, and empirically testing theoretically derived models (Creswell and Creswell, 2023). Within sport psychology, it has been argued that examining only the direct relationships among psychological variables may be insufficient to explain the mechanisms underlying athletes' psychological functioning. Consequently, contemporary research increasingly adopts integrative analytical models that not only investigate whether psychological variables are associated but also clarify the mechanisms through which these relationships emerge and the individual characteristics under which they vary. Mediation and moderation analyses provide a comprehensive framework for explaining psychological processes by offering a more nuanced understanding of the relationships among variables (Gürbüz, 2021). The present study assumed that the association between sport mindfulness and athlete burnout could be explained through athlete self-compassion. Specifically, higher levels of sport mindfulness were expected to foster a more understanding, supportive, and accepting attitude toward oneself, thereby enhancing athlete self-compassion. In turn, greater self-compassion was expected to serve as a protective psychological resource that reduces athlete burnout. However, the protective effect of athlete self-compassion on burnout was also expected to differ across individuals. In this regard, emotion regulation was considered an important psychological characteristic that may determine how effectively athletes utilize their self-compassion resources when facing stressful and demanding sport experiences. Accordingly, it was hypothesized that the strength of the relationship between athlete self-compassion and athlete burnout would vary depending on athletes' levels of emotion regulation. Furthermore, the indirect effect of sport mindfulness on athlete burnout was not expected to be identical for all athletes. More specifically, the mediating role of athlete self-compassion in the relationship between sport mindfulness and athlete burnout was hypothesized to differ according to athletes' emotion regulation capacities. This assumption is consistent with the moderated mediation framework, which proposes that mediation effects may vary under specific conditions. Accordingly, the proposed research model was developed as an integrated framework in which the relationship between sport mindfulness and athlete burnout is explained through athlete self-compassion, while the strength of this indirect relationship varies as a function of emotion regulation. The theoretical model tested in the present study is presented in Figure 1.

### Participants

Participants were recruited using criterion sampling, a purposive sampling technique that involves selecting individuals who meet predefined criteria relevant to the research objectives and is widely employed in behavioral science research (Patton, 2015). This sampling strategy was chosen based on the assumption that psychological constructs such as sport mindfulness, athlete self-compassion, emotion regulation, and athlete burnout can be more meaningfully and accurately evaluated by active athletes with sufficient sport-specific experience. The inclusion criteria were as follows: (a) being at least 18 years of age; (b) having actively participated in one of the Olympic combat sports (wrestling, boxing, taekwondo, judo, karate, or fencing) for a minimum of 1 year; and (c) voluntarily agreeing to participate in the study by providing written informed consent. Participants were excluded only if they were unable to maintain regular training and competition because of a serious injury or health condition. A total of 379 combat sport athletes who satisfied all eligibility criteria and met the assumptions required for the planned analyses were included in the study. Of these participants, 279 (73.6%) were men and 100 (26.4%) were women. Regarding competitive level, 114 athletes (30.1%) competed at the international (national team) level, whereas 265 athletes (69.9%) competed at the national level. According to sport discipline, the sample consisted of 132 wrestlers (34.8%), 87 taekwondo athletes (23.0%), 76 boxers (20.1%), and 84 athletes competing in judo, karate, or fencing (22.2%).

The adequacy of the sample size was evaluated using both theoretical and statistical criteria. First, the final sample exceeded the minimum recommended sample size based on a ±5% margin of error and a 95% confidence level. In addition, statistical power was verified using G^*^Power version 3.1.9.7 (Faul et al., 2007). The power analysis was conducted using the *F*-test family (Linear multiple regression: fixed model, *R*^2^ deviation from zero) with an assumed effect size of *f* ^2^ = 0.08, a significance level of α = 0.05, statistical power of 1–β = 0.95, and four predictor variables. The analysis indicated that a minimum sample of 223 participants was required. Because the present study included 379 participants, the sample size substantially exceeded this requirement, indicating that the study had sufficient statistical power to test the proposed moderated mediation model. Regarding the minimum values observed in sport-related characteristics, one participant reported zero weekly training sessions, 78 participants reported no competitions in the previous year, and 30 participants reported 1 year of sport participation. These values do not necessarily indicate the absence of sport involvement, as they may reflect temporary interruptions in training or competition participation due to seasonal transitions, competition scheduling, or other sport-related circumstances. Importantly, all participants met the predefined eligibility criterion of having at least 1 year of active participation in Olympic combat sports. Therefore, these participants were considered eligible for the present study because they had sufficient sport-specific experience to evaluate psychological constructs related to competitive sport participation, including athlete burnout and sport mindfulness. The demographic and sport-related characteristics of the participants are presented in Table 1.

**Table 1:** Demographic and sport-related characteristics of participants (*N* = 379).

Variable	Mean ±S.D.	Median	Min–Max
Age (years)	21.89 ± 5.83	20.00	18–43
Height (cm)	173.96 ± 9.27	175.00	150–197
Body mass (kg)	72.69 ± 16.57	73.00	40–130
Competition weight (kg)	69.97 ± 15.70	70.00	40–130
Weight reduction before competition (kg)	3.12 ± 2.79	3.00	0–20
Number of competitions in the previous year	3.57 ± 3.46	3.00	0–15
Years of sport participation	8.78 ± 5.26	8.00	1–31
Weekly training sessions	5.20 ± 2.74	5.00	0–16
Average training duration (min/session)	102.08 ± 26.17	90.00	60–180

### Data collection tools

#### Mindfulness Inventory for Sport (MIS)

Sport mindfulness was assessed using the Mindfulness Inventory for Sport (MIS) developed by Thienot et al. (2014), which was translated into Turkish and validated by Tingaz (2020). The Turkish version was adapted to assess mindfulness levels among university athletes. The instrument consists of 15 items rated on a 6-point Likert scale, ranging from 1 (“Almost Never”) to 6 (“Almost Always”). The scale comprises three dimensions: awareness, Non-Judgment, and Refocusing, with the items in the non-judgment subscale being reverse scored. Exploratory factor analysis conducted during the Turkish adaptation supported the original three-factor structure, which was subsequently confirmed through confirmatory factor analysis (CFA). The reported model fit indices were CMIN/DF = 1.84, RMSEA = 0.08, GFI = 0.86, CFI = 0.95, and IFI = 0.95. The Cronbach's alpha coefficients reported in the adaptation study were 0.81 for Awareness, 0.78 for Non-Judgment, and 0.77 for Refocusing. In the present study, Cronbach's alpha coefficients were 0.87, 0.90, and 0.89, respectively.

#### Athlete Burnout Questionnaire (ABQ)

Athlete burnout was measured using the Athlete Burnout Questionnaire (ABQ) developed by Raedeke and Smith (2001) and adapted into Turkish by Kelecek et al. (2016). The questionnaire comprises 13 items rated on a 5-point Likert scale, ranging from 1 (“Never”) to 5 (“Always”), with Item 12 reverse scored. The instrument includes three subscales: reduced Sense of Accomplishment, Emotional/Physical Exhaustion, and Sport Devaluation. The CFA conducted during the Turkish adaptation demonstrated a satisfactory three-factor structure (CMIN/DF = 1.73, RMSEA = 0.06, SRMR = 0.05, NFI = 0.92, NNFI = 0.94, CFI = 0.96, GFI = 0.92). The Cronbach's alpha coefficients reported in the adaptation study were 0.75 for Reduced Sense of Accomplishment, 0.87 for Emotional/Physical Exhaustion, and 0.83 for Sport Devaluation. In the present study, the corresponding reliability coefficients were 0.71, 0.89, and 0.84, respectively.

#### Self-Compassion Scale–Athlete Version–Short Form (SCS-AV-SF)

Athlete self-compassion was assessed using the Self-Compassion Scale–Athlete Version–Short Form (SCS-AV-SF) developed by Killham et al. (2018) and culturally adapted into Turkish by Tingaz and Atalay (2021). The scale consists of 12 items rated on a 5-point Likert scale and measures self-compassion as a single latent construct. Items 1, 4, 8, 9, 11, and 12 are reverse scored. The Turkish validation study supported the scale's construct validity, with CFA indicating acceptable model fit (CMIN/DF = 1.822, RMSEA = 0.063, SRMR = 0.058, GFI = 0.933, AGFI = 0.902). The adaptation study reported a Cronbach's alpha coefficient of 0.849.

#### Athlete Emotion Regulation Scale (AERS)

Emotion regulation was measured using the Athlete Emotion Regulation Scale (AERS). The original Emotion Regulation Questionnaire developed by Gross and John (2003) was translated into Turkish by Eldeleklioglu and Eroglu (2015), and subsequently adapted for athletes by Tingaz and Ekiz (2021). The athlete version consists of eight items rated on a 5-point Likert scale and includes two dimensions: suppression and Cognitive Reappraisal. The scale contains no reverse-scored items. The Turkish athlete adaptation demonstrated satisfactory construct validity, with CFA indicating good model fit (CMIN/DF = 1.617, RMSEA = 0.047, SRMR = 0.043, NFI = 0.930, TLI = 0.958, CFI = 0.972, GFI = 0.973, AGFI = 0.949). During the adaptation study, Cronbach's alpha coefficients were 0.653 for Suppression and 0.739 for Cognitive Reappraisal. In the present study, these coefficients increased to 0.71 and 0.83, respectively.

### Measurement model evaluation

To evaluate the construct validity of the measurement instruments, CFA was conducted. Second-order CFA models were specified for multidimensional instruments, whereas first-order CFA was applied for the unidimensional scale. Specifically, second-order CFA models were tested for the MIS, ABQ and AERS, whereas a first-order CFA model was tested for the SCS-AV-SF. This approach enabled the evaluation of whether the theoretically proposed factor structures adequately represented the observed data and whether the observed indicators appropriately reflected their corresponding latent constructs. Before conducting CFA, the assumption of multivariate normality was examined and found to be satisfied. Accordingly, the Maximum Likelihood (ML) estimation method, one of the most widely used estimation procedures in confirmatory factor analysis, was employed. Model fit was evaluated using the following goodness-of-fit indices: CMIN/DF (Chi-square/Degrees of Freedom), GFI (Goodness-of-Fit Index), CFI (Comparative Fit Index), and RMSEA (Root Mean Square Error of Approximation). The interpretation of these indices was based on the acceptable fit criteria recommended by Byrne (2016) and Gürbüz (2021). Convergent validity was assessed using the Average Variance Extracted (AVE) and Composite Reliability (CR) indices. Following the recommendations of Fornell and Larcker (1981), AVE values of 0.50 or higher indicate adequate convergent validity. However, when AVE falls below this threshold, convergent validity may still be considered acceptable if the corresponding CR exceeds 0.70. Furthermore, AVE values above 0.40, together with satisfactory CR values, have also been considered indicative of acceptable measurement quality (Maruf et al., 2021). Therefore, convergent validity was evaluated using these criteria in combination rather than relying on a single indicator. Internal consistency reliability was assessed using Cronbach's alpha (α) coefficients. The interpretation of reliability coefficients followed the guidelines proposed by George and Mallery (2019), whereby alpha values of 0.80 or above indicate good internal consistency and values of 0.90 or above indicate excellent reliability. Nevertheless, alpha coefficients between 0.60 and 0.80 were considered acceptable (Kalayci, 2018). The CFA results, model fit indices, standardized factor loadings, Cronbach's alpha coefficients, skewness and kurtosis statistics, together with the AVE and CR values for all measurement instruments, are presented in Table 2.

**Table 2:** CFA results, reliability coefficients, convergent validity indicators, and descriptive statistics of the measurement instruments.

Index	Good fit	Acceptable	MIS	ABQ	SCS-AV	AER
χ^2^/df	< 3	< 3(χ^2^/df) < 5	3.076	4.994	4.998	3.464
GFI	>0.95	>0.90	0.921	0.901	0.918	0.963
CFI	>0.95	>0.90	0.956	0.925	0.900	0.963
RMSEA	< 0.05	< 0.08	0.074	0.079	0.080	0.080
Cronbach's α	0.90	0.60-0.80	0.943	0.919	0.737	0.838
Skewness	-	−1/+1	−0.982	−0.319	−0.338	0.125
Kurtosis	-	−1/+1	0.663	−0.733	−0.049	0.250
AVE	>0.50	>0.40	0.616	0.500	0.410	0.461
CR	>0.70	>0.70	0.959	0.920	0.883	0.869

Although the χ^2^/df (4.994) and RMSEA (0.079) values for the Athlete Burnout Questionnaire (ABQ) were close to the upper limits of the acceptable thresholds, they remained within the recommended criteria. Therefore, the three-factor structure of the ABQ was considered adequately supported in the present sample.

### Ethical approval and data collection procedure

Ethical approval for this study was obtained from the Çanakkale Onsekiz Mart University Health Sciences Ethics Committee (Approval Date: February 18, 2026; Approval No.: 03/27; Project No.: 2026-YÖNP-0045). Participation was entirely voluntary, and written informed consent was obtained from all participants prior to data collection. Before completing the survey, participants were informed about the purpose, scope, and procedures of the study and were advised that they could withdraw from the study at any time without providing a reason. All study procedures were conducted in accordance with the ethical principles governing research involving human participants and complied with the principles of the Declaration of Helsinki. Data were collected online using an electronic questionnaire specifically prepared for this study and distributed to eligible participants.

### Data analysis

All statistical analyses were conducted using IBM SPSS Statistics version 25.0, AMOS version 21.0, and Hayes' PROCESS Macro. Before the primary analysis, the dataset was screened for missing data, outliers, and potential violations of statistical assumptions. Multivariate outliers were identified using Mahalanobis distance values. The assumption of univariate normality was evaluated based on skewness, kurtosis, and standardized univariate Z-scores. Skewness and kurtosis values were within the acceptable range of ±1 (Hair et al., 2013), and standardized univariate Z-scores were within ±3 (Kalayci, 2018), indicating that the data satisfied the normality assumptions required for parametric analyses. Pearson product-moment correlation analysis was used to examine the relationships among the study variables. Scatterplots were inspected to evaluate the linearity assumption, and no evidence of substantial non-linear relationships was observed. Multicollinearity was assessed using Tolerance and Variance Inflation Factor (VIF) values. Tolerance values greater than 0.20 and VIF values below 10 indicated that multicollinearity was not a concern. The construct validity of the measurement instruments was evaluated through CFA conducted in AMOS 21.0. Following confirmation of the measurement models, the proposed hypotheses were tested using Hayes (2022) PROCESS Macro. First, a mediation analysis was performed to examine the mediating role of SCS-AV in the relationship between MIS and ABQ. Second, a moderation analysis was conducted to determine whether AER moderated the association between SCS-AV and ABQ. Finally, a moderated mediation analysis was performed to investigate whether the indirect effect of MIS on ABQ through SCS-AV varied as a function of AER. Specifically, the study examined whether the mediating effect of SCS-AV differed across different levels of AER. The significance of the indirect and conditional indirect effects was evaluated using the bootstrap confidence interval method based on 5,000 bootstrap resamples (Hayes, 2022). Indirect effects were considered statistically significant when the 95% bootstrap confidence interval did not include zero (Hayes et al., 2017; Preacher and Hayes, 2008). Statistical significance was established at *p* < 0.05 and *p* < 0.01 for all analyses.

### Common method bias assessment

To evaluate the potential influence of common method bias (CMB), Harman's single-factor test was performed (Podsakoff et al., 2024). All measurement items included in the study were subjected to an exploratory factor analysis using Principal Component Analysis (PCA). The analysis identified 10 factors with eigenvalues greater than 1, indicating that the measurement items did not load onto a single latent factor. When a single-factor solution was examined, the first factor accounted for 24.37% of the total variance. Previous research has suggested that common method bias may represent a serious concern when a single factor explains more than 50% of the total variance (Eichhorn, 2014). The variance explained by the first factor in the present study was substantially below this threshold, suggesting that common method bias was unlikely to pose a significant threat to the study findings. In addition, the correlation coefficients among the study variables were examined, with the highest observed correlation being *r*= −0.36. It has been argued that severe common method bias is typically reflected by excessively high correlations among variables (e.g., *r* > 0.90) (Podsakoff et al., 2024). The relatively modest correlations observed in the present study further indicate that the likelihood of spurious associations attributable to common method bias was low. Taken together, the findings from Harman's single-factor test and the correlation matrix suggest that common method bias was not of sufficient magnitude to substantially influence the principal findings of the present study.

## Results

### Correlation analysis and descriptive statistics

Research findings regarding the Pearson correlation coefficients among the study variables are presented in Table 3. The results demonstrate the relationships among MIS, ABQ, SCS-AV, and AER. The findings revealed a significant negative correlation between MIS and ABQ (*r* = −0.15, *p* < 0.01), indicating that higher levels of MIS were associated with lower levels of ABQ. MIS was also positively and significantly associated with SCS-AV (*r* = 0.34, *p* < 0.01), suggesting that athletes with greater mindfulness tended to report higher levels of self-compassion. Likewise, a significant positive correlation was observed between MIS and AER (*r* = 0.32, *p* < 0.01). ABQ was negatively and significantly correlated with athlete SCS-AV (*r* = −0.36, *p* < 0.01), indicating that higher levels of SCS-AV were associated with lower burnout. Similarly, ABQ showed a significant negative relationship with AER (*r* = −0.31, *p* < 0.01), suggesting that athletes with stronger AER abilities tended to report lower burnout levels. Finally, SCS-AV was positively and significantly correlated with AER (*r* = 0.33, *p* < 0.01), indicating that greater SCS-AV was associated with more effective AER.

**Table 3:** Correlations between variables and descriptive analyses.

Variables	MIS	ABQ	SCS-AV	AER	Mean	S.D.
MIS	1				71.98	12.51
ABQ	−0.15^**^	1			37.44	12.81
SCS-AV	0.34^**^	−0.36^**^	1		36.20	5.58
AER	0.32^**^	−0.31^**^	0.33^**^	1	39.22	9.33

^**^p < 0.01.

The results of the mediation analysis examining the mediating role of athlete SCS-AV in the relationship between MIS and ABQ are presented in Table 4. The findings indicated that MIS positively and significantly predicted SCS-AV (*B* = 0.155, *p* < 0.001), suggesting that higher levels of MIS were associated with greater SCS-AV. In the second stage of the mediation model, the effect of SCS-AV on ABQ was examined. The results demonstrated that athlete SCS-AV negatively and significantly predicted ABQ (*B* = −0.798, *p* < 0.001), indicating that higher levels of SCS-AV were associated with lower levels of ABQ. The direct effect of MIS on ABQ was subsequently examined. After athlete SCS-AV was included in the model, the direct effect of MIS on ABQ was no longer statistically significant (*B* = −0.040, *p* = 0.452). In contrast, the total effect of MIS on ABQ remained statistically significant (*B* = −0.163, *p* = 0.002). The bootstrap analysis further demonstrated a significant indirect effect of MIS on ABQ through athlete SCS-AV [*B* = −0.124, BootSE = 0.026, 95% Boot CI (−0.1782, −0.0781)]. Because the 95% bootstrap confidence interval did not include zero, the indirect effect was considered statistically significant. Overall, these findings indicate that, after athlete self-compassion was included in the model, the direct effect of MIS on ABQ was no longer statistically significant, whereas the indirect effect remained significant. This pattern suggests that the association between MIS and ABQ operates primarily through athlete self-compassion, highlighting it as a key psychological mechanism linking sport mindfulness to athlete burnout.

**Table 4:** PROCESS model 4 mediation analysis results.

Path	*B*	SE	*T*	*P*	LLCI	ULCI	*R* ^2^
MIS → SCS-AV (a path)	0.155	0.022	7.187	< 0.001^**^	0.112	0.197	0.121
MIS → ABQ (c′ path)	−0.040	0.053	−0.753	< 0.452	−0.142	0.063	0.132
SCS-AV → ABQ (b path)	−0.798	0.118	−6.794	< 0.001^**^	−1.029	−0.567	—
Total effect (MIS → ABQ)	−0.163	0.052	−3.136	< 0.002^**^	−0.265	−0.060	0.025
Indirect effect (MIS → SCS-AV → ABQ)	−0.124	0.026	—	—	−0.178	−0.078	—

Unstandardized regression coefficients (B) are reported. MIS, Sport Mindfulness; SCS-AV, Athlete Self-Compassion; ABQ, Athlete Burnout. Indirect effects were evaluated using 5,000 bootstrap samples, and statistical significance was determined based on 95% bootstrap confidence intervals that did not include zero. ^**^p < 0.01.

**Figure 2:**
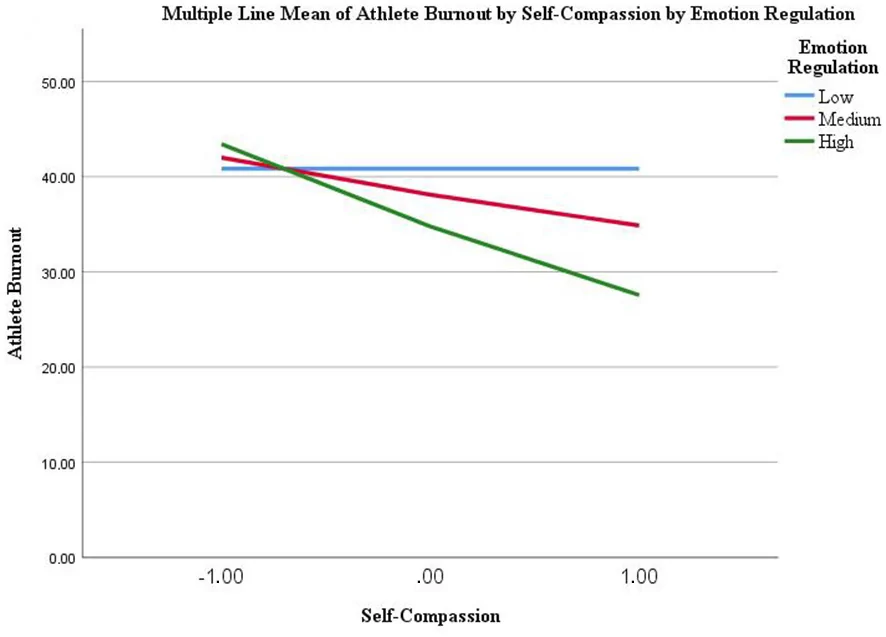
Interaction effect of emotion regulation on the relationship between athlete self-compassion and athlete burnout.

The results of the moderation analysis examining the moderating role of AER in the relationship between SCS-AV and ABQ are presented in Table 5. The findings indicated that the proposed moderation model was statistically significant and explained 23.1% of the variance in ABQ (*R*^2^ = 0.231, *p* < 0.001). Examination of the regression coefficients revealed that athlete SCS-AV had a significant negative effect on ABQ (*B* = −0.555, *p* < 0.001), indicating that higher levels of SCS-AV were associated with lower levels of ABQ. Similarly, AER also exhibited a significant negative effect on ABQ (*B* = −0.256, *p* < 0.001), indicating that athletes with higher levels of AER reported lower levels of burnout. Most importantly, the interaction term between SCS-AV and AER significantly predicted ABQ (*B* = −0.060, *p* < 0.001). The inclusion of the interaction term produced a significant increase in the explained variance (*R*^2^ = 0.059, *p* < 0.001), indicating that AER significantly moderated the relationship between SCS-AV and ABQ. To further examine the nature of this moderating effect, a simple slope analysis was conducted, and the results are presented in Figure 2. The analysis showed that at low levels of AER, the association between SCS-AV and ABQ was not statistically significant (*B* = −0.001, *p* = 0.998). In contrast, at the mean level of AER, SCS-AV was significantly and negatively associated with ABQ (*B* = −0.541, *p* < 0.001). Moreover, at high levels of AER, this negative association became substantially stronger (*B* = −1.202, *p* < 0.001). Taken together, these findings indicate that the protective effect of SCS-AV against ABQ becomes progressively stronger as athletes AER capacity increases. In other words, SCS-AV appears to play a more pronounced protective role against burnout among athletes with higher levels of AER.

**Table 5:** Moderating effect of emotion regulation on the relationship between athlete self-compassion and athlete burnout.

Predictor	*B*	SE	*t*	*P*	LLCI	ULCI
Constant	38.481	0.611	62.980	< 0.001^**^	37.280	39.683
SCS-AV	−0.555	0.112	−4.950	< 0.001^**^	−0.775	−0.334
AER	−0.256	0.066	−3.860	< 0.001^**^	−0.386	−0.126
SCS-AV × AER	−0.060	0.011	−5.368	< 0.001^**^	−0.082	−0.038
**Interaction effect test (SCS-AV** × **AER)**	*R* ^2^		**df1**	**df2**	***P***	
	0.059		1	375	0.001	
AER level	Effect	SE	t	p	LLCI	ULCI
Low (-1 SD)	−0.001	0.166	−0.003	< 0.998	−0.326	0.325
Medium (Mean)	−0.541	0.113	−4.809	< 0.001^**^	−0.762	−0.320
High (+1 S.D.)	−1.202	0.149	−8.082	< 0.001^**^	−1.494	−0.909

Unstandardized regression coefficients (B) are reported. SCS-AV, Athlete Self-Compassion; AER, Emotion Regulation; ABQ, Athlete Burnout. Continuous variables were mean-centered before analysis. Conditional effects were examined at low (−1 S.D.), medium (mean), and high (+1 S.D.) levels of the moderator. ^**^p < 0.01.

A moderated mediation analysis was conducted using PROCESS Model 14 to examine whether the indirect effect of MIS on ABQ through athlete SCS-AV varied according to the level of AER. The results are presented in Table 6. In the first stage of the model, MIS positively and significantly predicted SCS-AV (*B* = 0.155, *p* < 0.001), indicating that higher levels of MIS were associated with greater SCS-AV. In the subsequent model predicting ABQ, SCS-AV had a significant negative effect on ABQ (*B* = −0.555, *p* < 0.001). AER also exerted a significant negative effect on ABQ (*B* = −0.256, *p* < 0.001). Importantly, the interaction between SCS-AV and AER, which constitutes the core component of the moderated mediation model, was statistically significant (*B* = −0.060, *p* < 0.001). The inclusion of the interaction term resulted in a significant increase in explained variance (*R*^2^ = 0.059, *p* < 0.001), indicating that the relationship between SCS-AV and ABQ differed according to athletes' levels of AER. Examination of the conditional indirect effects revealed that the indirect effect of MIS on ABQ through SCS-AV was not statistically significant at low levels of AER (Effect = −0.000). In contrast, the indirect effect became significant at the mean level of AER (Effect = −0.084) and was stronger at high levels of AER (Effect = −0.187). Furthermore, the index of moderated mediation indicated that AER significantly moderated the conditional indirect effect (Index = −0.009). This finding demonstrates that the indirect relationship between MIS and ABQ through SCS-AV varies as a function of athletes' AER capacity. Overall, these findings suggest that the effect of MIS on ABQ is mediated by SCS-AV, and that the strength of this indirect effect depends on athletes' AER levels. Specifically, the protective role of SCS-AV in reducing burnout is more pronounced among athletes with higher levels of AER.

**Table 6:** Moderated mediation analysis of the indirect effect of sport mindfulness on athlete burnout through athlete self-compassion at different levels of emotion regulation.

Path	*B*	SE	*t*	*P*	LLCI	ULCI
MIS → SCS-AV (a path)	0.155	0.022	7.187	< 0.001	0.113	0.198
SCS-AV → ABQ (b path)	−0.555	0.117	−4.766	< 0.001	−0.785	−0.326
AER → ABQ	−0.256	0.068	−3.754	< 0.001	−0.391	−0.122
SCS-AV × AER	−0.060	0.011	−5.355	< 0.001	−0.082	−0.038
MIS → ABQ (direct effect)	0.002	0.051	0.031	< 0.975	−0.099	0.102
Index of moderated mediation						
**Moderator**	**Index**		**BootSE**		**BootLLCI**	**BootULCI**
Emotion regulation	−0.009		0.002		−0.015	−0.006
Conditional indirect effects of sport mindfulness on athlete burnout through athlete self-compassion						
**Emotion regulation level**	**Effect**		**BootSE**		**BootLLCI**	**BootULCI**
Low	−0.000		0.026		−0.049	0.050
Medium	−0.084		0.022		−0.134	−0.047
High	−0.187		0.038		−0.278	−0.124

Model statistics: Mediator model: *R*^2^= 0.121, F (1, 377) = 51.654, *p* < 0.001; Outcome model: *R*^2^ = 0.231, F (4, 374) = 28.063, *p* < 0.001; Interaction effect: *R*^2^ = 0.059, F (1, 374) = 28.679, *p* < 0.001.

## Discussion

The present study investigated the psychological mechanisms underlying the relationship between MIS and ABQ among Olympic combat sport athletes, as well as the conditions under which these mechanisms may vary. The findings indicate that the association between MIS and ABQ cannot be explained solely by a direct relationship. Rather, this association operates through an indirect pathway mediated by SCS-AV, and the strength of this indirect process varies according to athletes' levels of AER. In particular, the positive association between MIS and SCS-AV, together with the negative association between SCS-AV and ABQ, highlights the importance of psychological resources for athletes competing under intense performance demands. Furthermore, the moderating role of AER suggests that ABQ should not be viewed as the outcome of a single psychological characteristic but rather as the result of the interaction among mindfulness, self-directed attitudes, and emotional regulatory capacity.

The present study found a significant negative association between MIS and ABQ. This association reflects the bivariate relationship and total effect observed between MIS and ABQ; however, the direct effect of MIS on ABQ became non-significant after SCS-AV was included in the mediation model, indicating that this relationship was primarily explained through athlete self-compassion. This finding suggests that athletes' capacity to attend to and accept their present-moment experiences may be associated with lower levels of psychological exhaustion under conditions of intense performance pressure. In individual combat sports such as wrestling, boxing, judo, and taekwondo, athletes bear direct responsibility for competitive outcomes, making performance errors and failures more likely to be interpreted as personal shortcomings. Consequently, the manner in which athletes appraise stressors and respond to challenging experiences represents an important psychological process in understanding ABQ.

According to mindfulness-based perspectives, reducing individuals‘ tendency to evaluate experiences automatically and negatively promotes more flexible coping responses to stressful situations (Birrer et al., 2021; Wang et al., 2023). From the perspective of Lazarus and Folkman (1984) Stress and Coping Theory, the impact of stress depends not only on the stressful event itself but also on how the individual appraises that event. Within this framework, MIS can be considered a psychological resource that is associated with more adaptive evaluations of stressors such as performance pressure, failure, and physical demands, which may be related to lower levels of burnout. The present findings are consistent with previous studies demonstrating a negative association between MIS and ABQ. Research involving elite athletes has consistently shown that higher levels of mindfulness are associated with lower burnout, greater psychological wellbeing, and more effective stress management (Zhang et al., 2021; Myall et al., 2023). Likewise, mindfulness-based interventions have been reported to reduce stress, anxiety, and psychological distress while strengthening self-regulatory processes among athletes (García-Pérez et al., 2026; Noetel et al., 2019; Si et al., 2024; Su et al., 2024). Similarly, Campo et al. (2026) emphasized both the physical and psychological benefits of mindfulness training and advocated its integration into athletes' regular training programs. In addition, mindfulness has been identified as an important mechanism for regulating negative emotional responses among elite athletes (Solmaz and Yarayan, 2025). Collectively, these findings support the theoretical expectation of a negative relationship between MIS and ABQ. However, the results of the present study further indicate that the effect of MIS on ABQ cannot be explained solely by a direct association. Instead, this relationship should be understood within an integrated framework that incorporates both SCS-AV and AER.

The findings further demonstrated that athlete SCS-AV significantly mediated the relationship between MIS and ABQ. This result suggests that the protective effect of mindfulness on burnout is not transmitted directly but rather operates through the compassionate and supportive attitudes athletes develop toward themselves. In other words, athletes with higher levels of mindfulness appear to adopt more adaptive self-evaluative processes when confronted with stressful performance experiences.

This finding can be interpreted within the framework of Neff's (2003) Self-Compassion Theory. Self-compassion refers to an individual's ability to respond to failure, inadequacy, and other challenging experiences with kindness and understanding, to recognize such experiences as part of the shared human condition, and to maintain a balanced perspective toward negative thoughts and emotions. Theoretically, mindfulness provides the psychological foundation for the development of self-compassion by reducing excessive identification with adverse experiences. Consequently, the positive association observed between MIS and SCS-AV is consistent with the fundamental assumptions of self-compassion theory. The potential protective role of SCS-AV against burnout is particularly relevant in high-performance sport, where it serves as an important psychological resource under conditions of intense competitive pressure (Einarsdóttir et al., 2026; Glandorf et al., 2024). In Olympic combat sports, stressors such as competitive failure, performance errors, weight management, and intense competition may substantially influence how athletes evaluate themselves. Athletes with higher levels of self-compassion are less likely to interpret failures as indicators of personal inadequacy and appear better able to maintain psychological adjustment in the face of adversity (Kuchar et al., 2023). These findings are consistent with previous research demonstrating that SCS-AV is associated with lower burnout and greater psychological wellbeing (Ferrari et al., 2019). Accordingly, the present study extends the existing literature by identifying SCS-AV as a key psychological mechanism through which MIS contributes to lower levels of ABQ.

The present findings also demonstrated that AER significantly moderated the relationship between SCS-AV and ABQ, with the protective effect of self-compassion becoming stronger at higher levels of AER. Furthermore, the moderated mediation analysis revealed that the indirect effect of MIS on ABQ through SCS-AV varied according to athletes' AER capacities. These findings suggest that the effectiveness of athletes' psychological resources depends, at least in part, on their ability to regulate emotional experiences. The conditional effects indicated that the association between SCS-AV and ABQ was not significant among athletes with low levels of AER. This finding suggests that the protective influence of self-compassion may be constrained when athletes have limited capacity to regulate intense emotional experiences. In highly demanding contexts such as Olympic combat sports, athletes frequently encounter rapid emotional fluctuations following performance errors, competitive pressure, and defeat. Although self-compassion may promote a more accepting and supportive self-attitude, effective emotion regulation may be necessary for athletes to translate this adaptive mindset into effective coping responses during stressful situations. Therefore, AER may function as an important psychological capacity that is associated with athletes' ability to utilize the potential benefits of self-compassion in relation to burnout.

This result can be interpreted within the framework of Gross's (1998) Process Model of Emotion Regulation. According to this model, the ways in which individuals perceive, evaluate, and regulate their emotional experiences shape their responses to stressful situations. In competitive sport, where athletes frequently encounter emotionally demanding experiences such as performance errors, failure, and competitive pressure, effective AER may be associated with a greater capacity to utilize their psychological resources under demanding conditions. Consequently, AER may function as a complementary process that enhances the protective influence of self-compassion against burnout.

The present findings are consistent with previous studies demonstrating that AER is closely associated with psychological adjustment, stress management, and burnout among athletes. Recent evidence indicates that adaptive AER strategies are related to lower burnout and greater psychological wellbeing, whereas difficulties in AER may increase psychological distress in athletic populations (Fenton et al., 2025; Lane et al., 2012; Wagstaff, 2014; Wagstaff et al., 2025). Similarly, research involving elite athletes has identified AER capacity as an important psychological resource that promotes resilience under demanding performance conditions (Tamminen et al., 2025). Within the context of Olympic combat sports, athletes are continually exposed to substantial emotional demands arising from competition pressure, defeat, weight management, and high performance expectations. Under these circumstances, self-compassion may foster a more supportive and accepting attitude toward oneself, whereas effective AER may enable athletes to sustain this adaptive mindset during stressful situations. Overall, the present study suggests that ABQ is best understood as the outcome of the dynamic interplay among MIS, SCS-AV, and AER, rather than as the consequence of any single psychological factor.

### Strengths, limitations, and future directions

#### Strengths

One of the major strengths of the present study is that it examined not only the direct relationship between MIS and ABQ among Olympic combat sport athletes but also the underlying psychological mechanisms and the conditions under which these mechanisms may vary within an integrated conceptual framework. Specifically, the study provides a more comprehensive understanding of ABQ by integrating the theoretical perspectives of mindfulness, self-compassion theory and AER. This framework enabled the examination of the proposition that MIS may foster a more accepting and supportive attitude toward athletes' own experiences, while SCS-AV may function as a protective psychological resource under conditions of intense performance pressure. Furthermore, conceptualizing AER as a moderating factor contributes to a better understanding of how athletes utilize their psychological resources when confronted with stressful competitive situations. Another important strength of the study is the integration of mediation, moderation, and moderated mediation analyses within a single analytical framework. This methodological approach extends beyond identifying associations among variables by clarifying the psychological mechanisms through which these relationships operate and the individual characteristics under which they become stronger or weaker. An additional strength is the inclusion of a relatively large sample comprising athletes from multiple Olympic combat sports. The participation of athletes from wrestling, boxing, taekwondo, judo, karate, and fencing enhances the relevance of the findings across different combat sport disciplines. Moreover, the adequacy of the sample size was supported through *a priori* power analysis, and the measurement models were validated using confirmatory factor analysis, thereby strengthening the methodological rigor of the study. Finally, the present study contributes to the literature by conceptualizing ABQ not merely as a consequence of individual inadequacy or performance outcomes but as the product of the dynamic interaction among MIS, SCS-AV and AER. From this perspective, the findings provide a more holistic understanding of the psychological processes that support resilience and sustainable performance among Olympic combat sport athletes.

#### Limitations

Several limitations should be considered when interpreting the findings of this study. First, the cross-sectional design precludes conclusions regarding the temporal ordering of the variables and limits causal inference. Although the proposed model offers a theoretically grounded explanation of the relationships among MIS, SCS-AV, AER, and ABQ, the findings should be interpreted as correlational rather than causal. This is especially relevant for the mediation and moderated mediation results, as estimates of mediation obtained from cross-sectional data may not correspond to the underlying longitudinal parameters (Maxwell and Cole, 2007). Future longitudinal, experimental, and mixed-methods studies may provide a more comprehensive understanding of the dynamic relationships among these psychological constructs. Second, all data were obtained through self-report questionnaires. Although the measurement instruments demonstrated satisfactory psychometric properties, self-reported psychological characteristics may be influenced by factors such as social desirability and response bias. Future research would therefore benefit from incorporating multiple sources of information, including coach ratings, behavioral observations, and objective performance indicators, to provide a more comprehensive assessment of athletes' psychological functioning. Another limitation concerns the conceptualization of AER as a general psychological capacity. Future studies should examine specific AER strategies in greater detail, thereby providing a more nuanced understanding of the mechanisms through which AER influences ABQ. Finally, because the sample consisted exclusively of Olympic combat sport athletes, the generalizability of the findings to athletes from other sport disciplines, particularly team sports, or to athletes competing at different performance levels may be limited. Future studies involving diverse sporting contexts, cultural backgrounds, and competitive levels may help determine whether the relationships among MIS, SCS-AV, AER and ABQ operate similarly across different athletic populations.

#### Future directions

Future research should employ longitudinal and experimental research designs to further investigate the relationships among MIS, SCS-AV, AER and ABQ. In particular, intervention studies examining the effectiveness of mindfulness-based programs in enhancing SCS-AV, strengthening AER abilities, and reducing the risk of ABQ would provide valuable evidence regarding the causal mechanisms underlying these associations. Future investigations should also examine specific AER strategies, such as cognitive reappraisal and expressive suppression, to determine their differential contributions to ABQ. Furthermore, future studies should examine why the association between SCS-AV and burnout may become weaker or non-significant among athletes with lower AER capacity. Investigating whether difficulties in regulating intense emotional experiences limit athletes' ability to apply self-compassionate responses during stressful competitive situations may provide further insight into the boundary conditions of self-compassion-based approaches in sport contexts. Similarly, exploring the individual components of SCS-AV may help identify which dimensions provide the strongest protective effects in highly demanding sport environments. Another important direction for future research involves comparing athletes competing at different performance levels. Examining amateur, national-level, and international-level athletes may contribute to a better understanding of how psychological resources vary according to competitive demands and performance expectations. Finally, studies conducted across different sport disciplines and cultural contexts would provide broader insight into how MIS, SCS-AV and AER function within diverse sporting environments. Such research would enhance the generalizability of the current findings and contribute to the development of more targeted psychological interventions for athletes across a wide range of competitive settings.

## Conclusion

The present study examined the psychological mechanisms underlying the relationship between MIS and ABQ among Olympic combat sport athletes using an integrated conceptual framework. The findings indicate that the effect of MIS on ABQ cannot be explained solely by a direct association. Rather, this relationship operates through a multistage psychological process that is largely mediated by SCS-AV and varies according to athletes' AER capacity. The results indicate that higher levels of MIS were associated with higher levels of SCS-AV, consistent with a more accepting, understanding, and supportive attitude toward athletes' own experiences. In turn, SCS-AV appears to function as an important psychological resource associated with lower levels of ABQ. However, the protective effect of self-compassion was not equally evident across all athletes. Instead, AER emerged as a significant individual difference variable with the strength of this protective association varying across levels of AER. This finding suggests that the effectiveness of athletes' psychological resources depends not only on their availability but also on athletes' capacity to utilize these resources effectively under stressful performance conditions. The findings further demonstrate that ABQ in Olympic combat sports cannot be attributed solely to physical demands, training load, or performance expectations. Rather, burnout should be understood as a multidimensional psychological phenomenon arising from the interaction among MIS, self-directed attitudes, and AER processes. This perspective is particularly relevant in combat sports, where athletes are exposed to intense competitive pressure, a high cost of performance errors, and substantial individual responsibility. Under such conditions, interventions aimed at strengthening athletes' psychological resources may represent an important strategy for reducing burnout risk. Overall, the present study suggests that interventions designed to reduce ABQ should extend beyond traditional stress management approaches. A more comprehensive preventive strategy may be achieved by simultaneously promoting mindfulness skills, self-compassion-based practices, and AER capacity. By highlighting the complementary roles of these psychological processes, the present findings contribute to both the theoretical understanding and practical management of ABQ and offer valuable implications for supporting sustainable performance, psychological resilience, and long-term athletic career development among Olympic combat sport athletes.

## Data Availability

The raw data supporting the conclusions of this article will be made available by the authors, without undue reservation.
